# Flexible micromachined ultrasound transducers (MUTs) for biomedical applications

**DOI:** 10.1038/s41378-024-00783-5

**Published:** 2025-01-16

**Authors:** Sanjog Vilas Joshi, Sina Sadeghpour, Nadezda Kuznetsova, Chen Wang, Michael Kraft

**Affiliations:** 1 Department of Electrical Engineering (ESAT-MNS), KU Leuven, Belgium; 2 Leuven Institute for Micro- and Nanoscale Integration (LIMNI), KU Leuven, Belgium

**Keywords:** Sensors, Electrical and electronic engineering

## Abstract

The use of bulk piezoelectric transducer arrays in medical imaging is a well-established technology that operates based on thickness mode piezoelectric vibration. Meanwhile, advancements in fabrication techniques have led to the emergence of micromachined alternatives, namely, piezoelectric micromachined ultrasound transducer (PMUT) and capacitive micromachined ultrasound transducer (CMUT). These devices operate in flexural mode using piezoelectric thin films and electrostatic forces, respectively. In addition, the development of flexible ultrasound transducers based on these principles has opened up new possibilities for biomedical applications, including biomedical imaging, sensing, and stimulation. This review provides a detailed discussion of the need for flexible micromachined ultrasound transducers (MUTs) and potential applications, their specifications, materials, fabrication, and electronics integration. Specifically, the review covers fabrication approaches and compares the performance specifications of flexible PMUTs and CMUTs, including resonance frequency, sensitivity, flexibility, and other relevant factors. Finally, the review concludes with an outlook on the challenges and opportunities associated with the realization of efficient MUTs with high performance and flexibility.

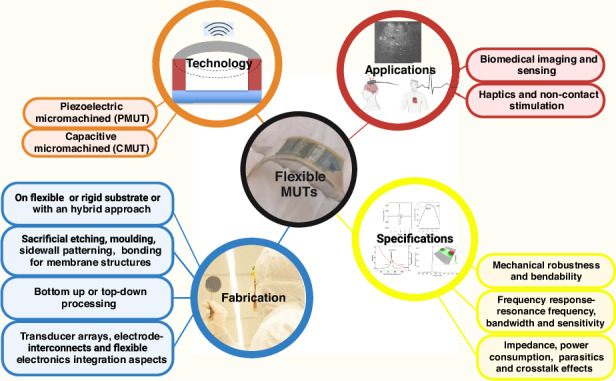

## Introduction

Recently, the healthcare and biomedical device market has seen a shift towards continuous monitoring. Flexible sensor technology has a significant potential to improve diagnostic quality by enabling continuous monitoring while in conformal contact with skin or tissue. This technology can reduce operator variability in medical diagnosis. Moreover, it presents a user-friendly solution due to its biocompatible interface with skin. As more healthcare services shift towards personalized medicine, adhesive smart patches for remote diabetic or heart patient monitoring (some key players are Abbott, Dexcom, iRhythm, and Medtronic) could be as commonplace as Fitbits and smartwatches. Devices like smart patches are made flexible using substrates such as elastomers, plastics, and hydrogels, allowing for stiction, stretching, or wrapping^1,2^. These devices are typically multilayered integrated systems consisting of flexible or stretchable substrates, active materials, electrodes-interconnects, electronics, and packaging materials, and their advantages such as being wearable, conformal, light, thin, without being fragile, and cost-effective for large-area applications, make them highly desirable^1,3,4^. This field is rapidly progressing with research and development, and various reports discuss the concepts, material-design challenges, and possible applications^5–9^. This review covers the progress made specifically in developing flexible micromachined ultrasound transducers (MUTs) for biomedical applications. MUTs are ultrasonic devices with membrane structures fabricated using micromachining.

Ultrasound technology has been extensively used in various fields such as nondestructive evaluation (NDE) and structural monitoring^10^, ultrasonic cleaning and material processing^11^, underwater communication^12^, particle and cell manipulation^13^, and object recognition^14^. Ultrasound is also desirable for low-power consumer applications with spatial bandwidth sharing due to its limited propagation distance in the air and unregulated sound spectrum beyond the audible range^15^. Moreover, ultrasound technology has been proven to be extremely useful in healthcare where especially, medical imaging based on rigid ultrasound probes stands out for diagnostics and disease prevention aspects^16^. However, continuous monitoring and biomedical sensing in mobile settings have been impossible due to the bulkiness of the rigid probes and instrumentation. In comparison with rigid counterparts, flexible ultrasound technology can offer added advantages due to its conformal contact and compact user-friendly design. These advantages have enabled the applications of flexible ultrasound transducers based on classical thick-film piezoelectric technology, such as continuous imaging of internal organs^1^, cardiac assessment^17^ including blood pressure monitoring^18^, and tissue elastography^19^. Conformal contact is also advantageous for a wide variety of applications such as retinal stimulation^20^, fetal heart monitoring^21^, and needle guidance procedures^22^. Moreover, flexible transducers allow mechanical focusing by shaping the device like a lens without costing energy for ultrasound focusing. This is extremely useful in neuromodulation^23^, bone injury treatment^24^, haptics^25^, and remote surgeries if combined with gesture recognition^26^. Furthermore, flexibility widens the field of view which comes in handy for Intravascular^27^ as well as transurethral^28^ ultrasound where the transducer can be cylindrically wrapped around a catheter.

Ultrasound transducers generate ultrasound waves utilizing acoustic pressure wave vibration^29^ using various methods such as the piezoelectric effect^30^, electrostatic actuation^31^, magnetostriction^32^, and the photoacoustic effect^33^. Conventional machining is typically used for the fabrication of ultrasound transducers involving non-cleanroom techniques^34^. In a classical piezoelectric transducer, the piezoelectric material is utilized as thick piezoelectric ceramic producing thickness mode vibration (also known as bulk piezoelectric technology)^35^. However, they face performance limitations arising mainly from associated fabrication techniques such as interelement pitch limitations introduced by mechanical dicing. For instance, an 8 megahertz (MHz) transducer operating in a tissue medium has a half-wavelength pitch requirement of less than 100 µm^36^. Besides array pitch, it is challenging to reduce the power consumption and form factor due to constraints imposed by bulky design. Furthermore, they have limited design flexibility because the resonance frequency is related only to the device’s thickness. Another foreseen concern is that most traditional materials used for these transducers are lead-based, which poses environmental hazards.

Nowadays, micromachining techniques play a crucial role in improving the fabrication of ultrasonic devices (MUTs) with considerable performance enhancement^37^. This enhancement can be attributed to small feature sizes, batch fabrication, and the possibility of integration with complementary metal oxide semiconductor (CMOS) technology. MUTs can be classified into two types. In a piezoelectric micromachined ultrasound transducer (PMUT), a piezoelectric thin film in the membrane actuates the device in flexural mode^38^. On the other hand, a MUT based on the electrostatic principle is called capacitive MUT or CMUT where the capacitor involves an air gap. These transducers also work in a flexural vibration mode with their vibrations set up with electrostatic forces between electrodes^39,40^. Also, note that there is a fundamental difference between piezoelectric and electrostatic transduction regarding the electromechanical coupling aspects. In the piezoelectric type of transducers, the typically low electromechanical coupling coefficients associated with the piezoelectric materials limit the energy conversion from the electrical to the mechanical domain. Hence, the piezoelectric material technology revolves around exploiting the energy density of the piezoelectric material to push the electromechanical coupling limit to a higher value^41–43^. However, CMUT membranes can theoretically couple all the energy resulting in an ultra-high bandwidth^44^. The reader is referred to the reported literature for further details on bulk piezoelectric transducers^29^, PMUTs^38^, and CMUTs^39,40^.

**Fig. 1:**
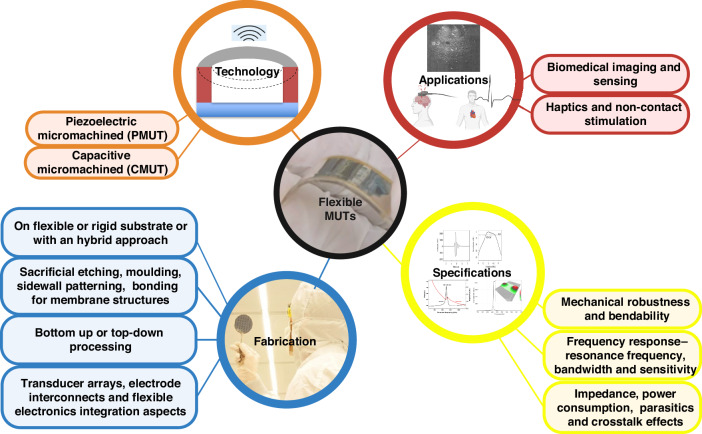
The landscape of flexible MUTs is covered in this paper. From technology and design-fabrication aspects to specifications and targeted applications

Although bulk piezoelectric transducers are also providing key insights for the technology advancement of MUTs regarding applications, array schemes, and interconnects, fundamentally, flexible MUT devices are fabricated based on the technology pillar of micromachining and MEMS processing. It is basically a PMUT or a CMUT fabrication with a membrane structure combined with various approaches to render them flexible, for instance, the assembly of an active piezoelectric such as a thin film of Lead Zirconate Titanate (PZT) on a polyimide substrate to fabricate a flexible PMUT^45^. Many more flexible PMUTs and CMUTs like these will be comprehensively examined in this paper covering their technology-fabrication processes, specifications, and potential applications as demonstrated in Fig. 1.

The paper is structured as follows: Next section provides an in-depth background on flexible MUT applications, highlighting the motivations behind the technology’s development. It also briefly details the specifications of flexible MUTs from both application and design perspectives. Furthermore, this background section discusses the substrates and material designs employed in fabrication of flexible MUT devices. Section “Flexible Micromachined Ultrasound Transducer Types” offers a comprehensive examination of the fabrication processes and summarizes the reported strategies for fabricating flexible MUTs, including PMUTs and CMUTs, along with their nuances. Section “Discussion, challenges, and outlook” outlines the key challenges and presents an outlook on future developments.

## Flexible MUT technology background

### Need for flexible MUTs and potential applications

**Fig. 2:**
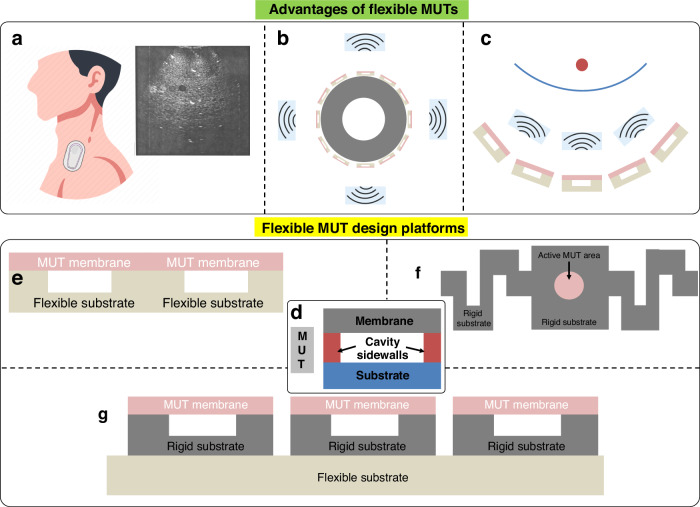
Flexible MUT background. **a** On the left, an illustration of a flexible ultrasound transducer patch conformally attached to the human neck^18^ and on the right, ultrasound imaging of Gammex 403GS phantom on the right (adapted with permission)^49^. **b** Schematic showing 360^o^ angular coverage of a cylindrical ultrasound transducer^2^. **c** Conceptual objective diagram for mechanical focusing using a flexible ultrasound transducer^57^. **d** Schematic of a MUT, which could be, a PMUT or a CMUT (both contain a membrane, a cavity, and a substrate with a difference in constituent layers). **e** MUTs on a flexible substrate^3,45^. **f** MUTs connected by springs, both fabricated out of rigid substrate^64^. **g** Rigid MUTs joined using flexible substrates^2,57^. Schematics are not drawn to scale

A potential application of flexible ultrasound is in biomedical imaging^46^. In ultrasound imaging, a propagating ultrasound wave (pulse) is partially reflected from the interface between different tissues, and the echo is measured as a function of time to form an image. A few things to consider while optimizing the transducer system for imaging are as follows: (1) For acquiring an image with a superior quality, the transducer should not exhibit ringing. In other words, it should have high bandwidth to effectively shorten the pulse duration and thus increase the imaging resolution. (2) The pulse duration can also be shortened by increasing operation frequency. (3) Additionally, the echo intensity drops drastically with the propagation distance or imaging penetration depth, also resulting in decreased signal-to-noise-ratio (SNR). The transducer is mounted either on the skin or placed inside the body depending on the target organ for imaging and transducer technology. Typically, if the target entities are situated close to the skin, the transducers can be mounted on the skin. In such cases, rigid ultrasound transducers^16,47^ with flat bases cannot achieve solid interfacial contact with irregular nonplanar surfaces, for instance during capturing movement of the knee while exercising. Air gaps at these interfaces lead to significant acoustic energy reflections and wave distortions, creating imaging artifacts^48^. The ultrasonic gel used to remove the air gaps causes significant canceling of small response echoes and also transmission loss. This problem can be solved by flexible ultrasound transducers, which can achieve conformal contact in an epidermal scheme (as shown in Fig. 2a-left) and has been demonstrated by various research groups. Wong et al. designed a flexible CMUT at 4 MHz for abdominal imaging^49^. They imaged a Gammex 403GS Phantom, which provided excellent visualization of the circular cyst models, and successfully reported liver and kidney images (Fig. 2a-right). In ultrasound imaging, phantoms are physical objects with a controlled medium for scanning used to test and calibrate ultrasound equipment. Furthermore, at a less mature stage, there are also a few more reported MUTs being developed for medical imaging of other targeted organs. A CMUT reported by Omidvar et al. can operate at up to 15 MHz targeted at musculoskeletal imaging^3^. The CMUT design can be particularly suited for visualizing anatomical structures situated within elongated and curved regions, such as limbs. They demonstrated pulse-echo functionality at 4 MHz. Joshi et al. reported a flexible PMUT patch around 4 MHz with a targeted application of tendon strain estimation in the knee^45^. Note that the relative motion between ultrasound image frames can be used to estimate the strains. Such flexible (MUT) transducers present a potential solution for continuous monitoring without obstacles, by serving as conformal patches that are comfortable and easily applicable. These wearable devices possess mechanical properties like skin and boast an ultrathin profile that adapts over time, enabling real-time data acquisition. It presents great possibilities such as tracking muscle activity during exercise sessions and transmitting information wirelessly. Moreover, large-area wearable ultrasound transducers can negate the need for expertise in manually scanning and positioning rigid ultrasound transducers.

To image internal body parts which are possibly difficult to image with a transducer on the skin, a catheter can be used to put the transducer inside the body. A prominent example is intravascular ultrasound imaging (IVUS) where flexible transducers cover a specially designed catheter, which is put in arteries^31,50^. Needless to say such applications require an extremely small bending radius, typically a millimeter. IVUS allows seeing the inner wall of blood vessels in living individuals, especially the arteries, where it is used to determine the amount of atheromatous plaque built up leading to stenosis or narrowing of the artery and in turn, heart attacks. The advantage of flexible transducers in such applications is that they can enable the transmission and detection of signals with increased angular coverage relative to rigid medical ultrasound probes. A cylindrical transducer can see a 360^o^ view, unlike a rigid planar transducer (see the illustration in Fig. 2b). Zhuang et al. demonstrated a conformal CMUT array with submillimeter bending radius for sideways wrapping a catheter which potentially could enhance the IVUS system to simplify diagnostic and surgical procedures^2^. Another study from Caronti et al. demonstrated pulse-echo characterization of a 10.7 MHz flexible CMUT with high flexibility and curvatures required in convex endocavity probes (scanning inside of the body for rectal or vaginal exams with shallow depth and wider field)^47,51^.

Flexible ultrasound transducers, with variable curvature and focal length within a specific range, function as a curved lens and provide mechanical focusing that reduces the power required for electronic focusing in phased array actuation (illustrated in Fig. 2c). This feature is particularly advantageous for haptics and non-contact stimulation applications. Haptics technology utilizes local ultrasound pressure fields to create virtual pressure objects that can stimulate nano-receptors in the fingertip after down-modulation to frequencies below 500 Hz. Interuniversity Microelectronics Centre (IMEC) has demonstrated display-compatible polymer-based large-area PMUTs at 400 kHz frequencies^52^. This effectively serves as carrier frequencies for mid-air haptic feedback at around 1 cm from the source when trading off resolution with attenuation. They recently demonstrated an acoustic twin-trap with mid-air micro-manipulation and levitation^53^ as well as a gesture recognition platform^54^. Combining haptics technologies with gesture recognition based on pulse-echo measurements could have interesting implications for remote surgeries. Beyond haptics, the ability to mechanically focus ultrasound with flexible transducers can be used for neuromodulation applications due to non-invasiveness, and strong penetration. Focused ultrasound technology can be used as a treatment for movement disorders, such as Parkinson’s disease by targeting specific brain regions and specifically, low-intensity, low-frequency ultrasound (LILFU) with carrier frequencies between 250 kHz and 500 kHz has been shown to modulate neuronal activity^55,56^. As a development in this direction, flexible PMUTs have shown promising acoustic pressures suitable for neuromodulation using low-intensity ultrasound^57^.

### Flexible MUT specifications

Because an ultrasound transducer is a resonant device, its performance is greatly dependent on its resonance behavior which can be understood from characteristics such as resonance frequency, bandwidth, and sensitivity. These specifications are determined by performing various characterizations based on targeted applications. For instance, to use a transducer for tissue imaging, a pulse-echo experiment can be performed underwater as the water medium has closer acoustic properties to the tissue medium^3^. The echo signal combines transmit and receive response and has information about the transducer resonance frequency and bandwidth. Furthermore, the combined (transmit and receive) sensitivity can be determined from the received signal amplitude in terms of signal attenuation. The signal attenuation directly affects the SNR. The transmit and receive sensitivities can also be measured individually in various ways. For instance, MUTs can be measured at the transducer surface with in-air transmit response recorded by a Laser Doppler Vibrometer (LDV). Transmit pressure sensitivity can also be noted at a targeted distance using a microphone in-air or hydrophone underwater, while receive sensitivity can be measured using a calibrated transducer that generates a pressure wave with a known amplitude that can be received at the device under test (DUT) and measured in terms of voltage (details in Ref. ^30^). Electrical impedance is another important specification of an ultrasound transducer that indicates its resonance frequency through the peak of the phase response^58^, allows the calculation of the coupling coefficient- an important metric of the efficiency of the transducer for electrical-mechanical energy conversion^59^, and is important for electronic instrumentation, impedance matching, and understanding the transducer power consumption^60^.

These performance metrics are the result of various interlinked design considerations involving structure geometry as well as materials and hence present a complex optimization problem. For instance, a factor that affects the transducer’s impedance is its intrinsic capacitance. The capacitance depends on the piezoelectric film thickness and dielectric constant for a PMUT whereas on the gap in a CMUT. Moreover, it is also related to the lateral dimensions of the transducer defined by geometry and array design. The lateral and longitudinal dimensions of the constituent layers along with material properties also affect the resonance frequency^30^. Furthermore, the interelement distance (pitch) between the cells or elements becomes critical as a design parameter in imaging applications as a half-wavelength pitch condition should be met to avoid side lobes while performing medical imaging^36^. Pitch together with an active transducer area defines one more parameter called fill factor which shows the effective area of the transducer affecting acoustic pressure^61^. One more key aspect of a flexible transducer design is its flexibility. A specific bending radius range is typically demanded by the application, and the transducer is mechanically designed to meet the bending radius criteria. The bending radius depends on the used materials and transducer assembly.

### Flexible MUT substrates and assembly

The schematic of a MUT is shown in Fig. 2d. It is marked by the presence of a membrane, a cavity, and a substrate. The substrate is typically the thickest part of the transducer assembly and affects the flexibility of the devices. On the other hand, active and structural materials for these transducers are typically thin films. Based on the substrate material flexibility and the way transducer elements are assembled three categories of flexible, meta-rigid, and hybrid platforms for the flexible MUTs can be explained. Figure 2e shows the most common flexible transducer design approach- transducer on a flexible substrate (referred to as ‘flexible substrate-based approaches’ in the next sections), which could be stretchable such as Polydimethylsiloxane (PDMS)^62^, or just bendable like polyimide^45^, polyethylene terephthalate (PET)^63^. In such designs, low-modulus materials like polymers make up most of the transducer to ensure local flexibility. High-modulus materials such as silicon, its compounds, other piezo-ceramics, metal foil substrates, or thin metal/ceramic films forming interconnects or active layers are typically not more than a few µm in thickness to maintain flexibility throughout. However, these devices suffer from low performance because of several reasons: 1) low modulus structural layers limit the resonance frequency of devices; 2) often these structures, are associated with higher material damping and hence strongly dampen the vibrations reducing the sensitivity. In contrast, the silicon MEMS technology has demonstrated quite promising performance. Sadeghpour et al. (design concept in Fig. 2f) demonstrated an approach for flexible ultrasound transducers using rigid silicon springs as flexible links^64^. This interesting way to modify a rigid structure to a flexible one by adding a spring as a compliant structure is referred to in this paper as meta-rigid approach where the link between the transducers constitutes compliant structures made out of stiff material. Moreover, the complete device processing can be done on a silicon wafer using standard micromachining techniques with the benefits of high performance, reliability, cost-efficiency, and the possibility of CMOS integration. However, fill factors and flexibility of the transducer array are limited due to complicated steps in the fabrication process, especially deep reactive ion etching (DRIE), and also the compliant link. In the context of array design, such a design allows for limited space for interconnects. This worth researching approach is recent in the literature and needs to be developed to solve the aforementioned problems. In a word, flexible substrates allow for better flexibility and possibilities for electronics integration while devices built on rigid silicon substrates tend to have better transducer performance metrics, such as transmit sensitivity.

The tradeoff between flexible substrate technologies and silicon MEMS can be partially overcome by the hybrid approach, which involves fabricating devices that are partly flexible and partly rigid^65^. This approach is becoming increasingly popular in flexible ultrasound technology, where both flexibility and high performance are important. In ultrasound transducers, the hybrid approach involves rigid transducer elements joined through flexible links (referred to as ‘hybrid approaches’ in the next sections as well). As an example, Lee et al. fabricated a silicon-based array of transducers and then bonded them to a thick PDMS matrix. Finally, they diced the array to separate each PMUT element (design concept in Fig. 2g)^57^. The transducer on the rigid silicon substrate showed high acoustic pressure while the link between the transducers with low Young’s modulus structures allowed the flexibility of the complete transducer array. Here also, low fill factors and electronics integration present challenges. Interconnects and electronics integration become easier in designs with a relatively flatter and continuous topography throughout the array which is easier in flexible substrate-based approaches.

## Flexible micromachined ultrasound transducer types

After discussing the basics of flexible transducer technology, we will discuss a detailed analysis of the technologies mentioned in the introduction. During the fabrication of MUTs, a membrane, and a cavity will be produced on a substrate as illustrated in Fig. 2d. For cavity fabrication, the following processes are typically performed: (1) subtractive processing of sidewall material where normally, dry etching, either reactive ion etching (RIE) or DRIE, laser machining or photo-patternable epoxy development processes are performed. This is common for PMUTs where it is usual to not have a separate substrate layer. (2) sacrificial layer etching where first, a sacrificial layer is patterned on a substrate. Then, membrane and cavity sidewalls are deposited. Finally, the sacrificial layer is etched to form a MUT cavity, which is a common method of CMUT fabrication. Beyond realizing a cavity, the flexible MUT fabrication can often involve more process steps, listed next: (3) bonding procedures of a membrane with the cavity sidewalls or bonding of cavity sidewall to a substrate or both. This can be achieved with a simple thin film coating or lamination or complicatedly, by transferring a layer realized on a temporary carrier, involving the carrier wafer bonding and release. (4) Release of flexible structures from temporary carriers (the use of rigid temporary carriers like silicon or glass is very common). Moreover, it is also possible to perform the above processes in reverse order where first, membrane structures followed by cavity sidewalls are realized on a temporary carrier in a top-down scheme. Optionally, they are bonded to a flexible substrate. Finally, the temporary carrier is released to complete the fabrication. A complete flexible MUT fabrication process flow will typically involve a combination of these processes.

### Piezoelectric micromachined ultrasound transducer (PMUT)

**Fig. 3:**
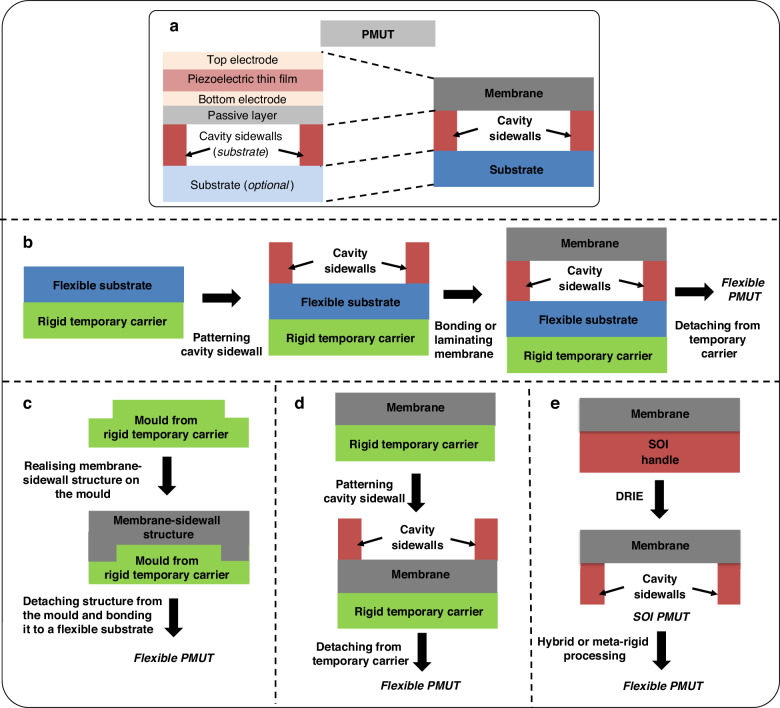
Overview of flexible PMUT fabrication. **a** Schematic of a PMUT^38^. **b** Bonding or lamination process^73^. **c** Molding for cavity fabrication in PMUTs followed by bonding to a flexible substrate^68^. **d** Top-down fabrication for PMUTs^45^. **e** SOI PMUTs followed by hybrid or meta-rigid processing^57,64^. The schematics are not drawn up to scale

**Fig. 4:**
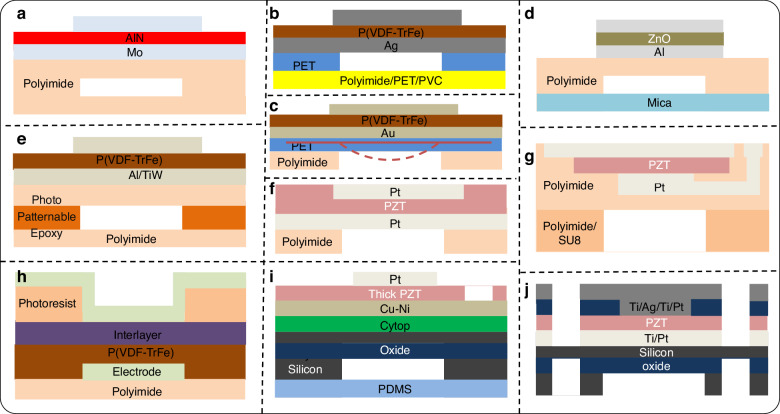
Overview of flexible PMUT designs. **a** Schematic of a PMUT realized using a sacrificial release and transfer process^66^. **b** Schematic of a PMUT realized using laser micromachining and TRT^63^. **c** Flexible PMUT with the membrane being curved via hot-processing^72^. **d** PMUT on Mica substrate using DRIE Silicon mold and adhesive bonding^68^. **e** Released PMUT fabricated using bottom-up processing on a single temporary carrier^73^. **f**–**h** Flexible PMUTs realized using a top-down process flow in combination with sacrificial membrane release, DRIE, and mechanical release, respectively^45,74,76,77^. **i**, **j** Hybrid and meta-rigid approaches on SOI wafers^57,64^

**Fig. 5:**
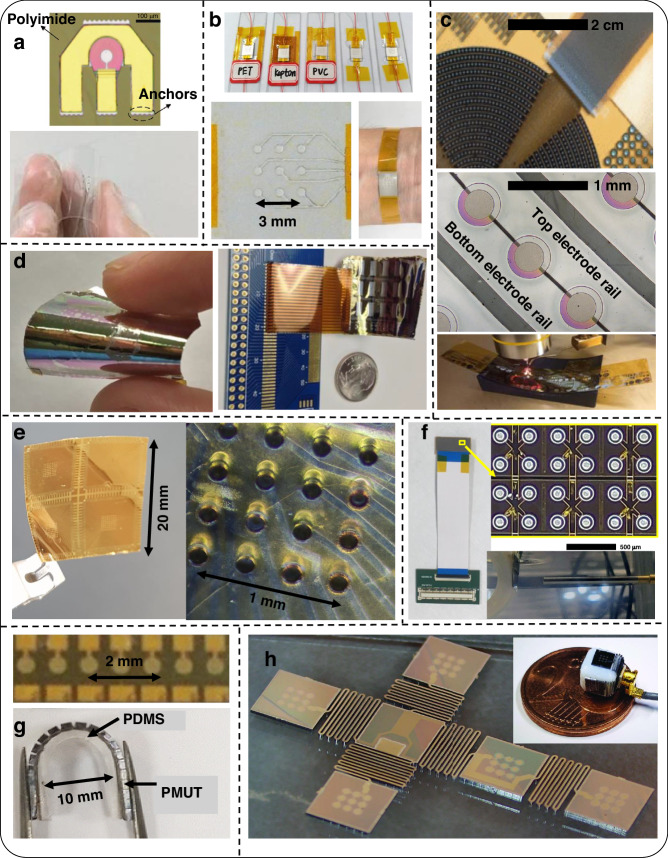
Overview of reported flexible PMUT devices. **a** Optical microscope image of flexible PMUT membrane transferred on a polyimide substrate (top) and released device (bottom)^66^. **b** PMUT assembled on various flexible substrates (top left), view of the patterned Ag top electrodes (bottom left), and device wrapped around the wrist (bottom right)^63^. **c** Polymer-based PMUTs fabricated at IMEC using Bottom-up processing. Image of the annular array (top), magnified image of a part using an optical microscope (middle), and flexible device being characterized (bottom)^53,73^ **d** Released PZT-based devices on polyimide using a top-down process, under bending (left) and with ACF connections (right)^76^. **e** Fully addressed high-frequency PZT-based flexible PMUT device on polyimide (left), on SU8 (middle)^45,77^. **f** On the left, RC addressed flexible PMUT connected to PCB using FFC. On the top right, optical microscope image of a part of the array, and at the bottom, the array being characterized while bending in water^83^. **g** Optical microscope image (top) and flexibility (bottom) of SOI-based hybrid PMUT array^57^. **h** SOI-based meta rigid PMUT array released and bent along a cube (inset). All pictures have been adapted with permission^64^

PMUT (schematic in Fig. 3a) consists of a membrane with a piezoelectric thin film and operates in flexural mode. The membrane typically contains a patterned layer stack of active layers (electrodes and piezo-electric thin film) along with a passive layer. The membrane is driven by applying an excitation voltage between the top and bottom electrodes. The applied electric field forms transverse stress in the active piezoelectric layer, which causes the membrane to displace out-of-plane, generating a pressure wave in the outer medium. The resonance frequency of the PMUT, because of the flexural mode, is mainly defined by the geometry and materials as they influence the mechanical stiffness and mass density of the membrane^59^. However, intrinsic stress in the membrane generated during fabrication can drastically affect the resonance frequency and this is one of the reasons why practical PMUTs usually perform differently than their theoretically expected performance. Also, this makes membrane structure fabrication challenging. The following discussion is about various approaches to fabricating flexible PMUTs. The fabrication processes are schematically shown in Fig. 3, whereas Figs. 4 and 5 present schematics and images of reported flexible PMUTs, respectively.

#### Flexible substrate-based approaches

Sun et al. reported a process for flexible aluminum nitride (AlN) PMUTs (see Fig. 3b for the fabrication schematic, Fig. 4a for the device schematic, and Fig. 5a for images of the fabricated device)^66^. The bottom electrode layer is used as the structural layer to reduce the input impedance. This could be advantageous for resonance behavior as well, but this could reduce the device flexibility meriting careful considerations of the design. During fabrication, the PMUT active layers were first fabricated on a temporary silicon carrier with an oxide sacrificial layer. Polyimide was deposited on another silicon carrier with polymethyl-methacrylate (PMMA) as a sacrificial layer. This polyimide serves as a flexible substrate and sidewall material for the flexible PMUT. The polyimide was etched for realizing sidewalls. The next step is to bond the membrane. To achieve this, another carrier, a PDMS stamp, was introduced to transfer the membrane on the sidewall structure. After the removal of the sacrificial layer, the structure was transferred to the PDMS stamp. The membrane was then aligned with a sidewall structure. At this point, the flexible PMUT is attached to a silicon carrier from one side and PDMS from the other. With an appropriate move rate, the device was separated from PDMS, and acetone was used to dissolve PMMA to release the device from the silicon. Note that, here, controlling adhesion forces between the donor, the structure, and the receiver is done through sacrificial layers, and additional surface treatments such as ultraviolet (UV) exposure, or oxygen (O_2_) plasma. Moreover, multiple carriers are involved which may impact the cost and convenience of the manufacturing process.

Studies by Liu et al. introduced thermal release tape (TRT) instead of sacrificial layers on a rigid carrier^63,67,68^. TRT is a tape with convertible adhesive force widely used for transferring graphene^69^. When it is heated to 90–100 ^o^C, the adhesive force will vanish irreversibly. This makes the transfer printing process simple. It takes out additional complex processes such as surface treatments in the case of PDMS stamps, and vias for sacrificial release, which improves the micromachining stability. However, TRT cannot support high-temperature fabrication. The PMUT fabrication demonstrated a one-side silver (Ag) coated commercial polyvinylidene fluoride (PVDF) sheet along with the silver pasted top electrode as a thick PMUT membrane (fabrication scheme in Fig. 3d)^67^. A PVDF film is used as the piezoelectric layer, because of its dielectric and piezoelectric properties, wide bandwidth, high sensitivity, optical transparency, and mechanical flexibility^70^. For patterning top Ag, laser-patterned polyimide tape (Kapton) is used as a shadow mask. This membrane assembly is inverted and pasted on a temporary carrier. Next, polyimide was spun on top of a PVDF film having two functions: passive and adhesion layer. A thick polyimide (Kapton) sheet patterned by laser micromachining served as a flexible substrate and cavity sidewalls. The substrate was aligned-bonded on PVDF film without extra pressure. Such fabrication process can be extended to the use of alternative materials for substrates and sidewalls such as PET (schematics in Fig. 4b and images of the fabricated array in Fig. 5b)^63^. The paper also demonstrated that a laser-processed substrate can be used as an adhesive. However, this fabrication lacks a passive layer which is instrumental in improving the sensitivity of the PMUT device by pushing the neutral axis away from the piezoelectric^71^. A recently published study, with a comparable schematic, revealed an alternative approach- curving the membrane as illustrated in Fig. 4c^72^. This adjustment led to a twofold increase in performance compared to the flat version. During fabrication, a multilayer membrane film, consisting of a PET passive layer, PVDF piezoelectric layer, and gold (Au) electrodes, is placed between a copper (Cu) casting mold and then hot-pressed for 5 minutes at 95 °C under a pressure of 2 bars. The alignment of active layers on the cavity sidewalls during the bonding process as demonstrated in previous designs is either prone to inaccuracies or needs specialized equipment which is especially critical for designs with exceedingly small features. Yet another work from Liu et al. demonstrated a PMUT based on flexible and transparent mica substrates (Fig. 3c shows the fabrication schematic and Fig. 4d shows device schematic)^68^. This avoided alignment during the bonding procedure and is achieved using a molding process, aligning membranes and sidewalls during molding, which simplifies the process of realizing the membranes. The fabrication started with a silicon mold, which defines the membrane cavity, formed by standard photolithography and DRIE. Then, the polyimide passive layer and zinc oxide (ZnO) piezoelectric sandwiched between Aluminum (Al) electrodes were deposited. In parallel, a thin layer of PDMS was spin-coated on the mica substrate. PDMS was partially cured to serve as the adhesive layer. Then the structures above the silicon mold were transferred to the mica substrate using TRT. After that, a thermal treatment was carried out to fully cure the adhesive layer and to peel off the TRT simultaneously. Finally, mica was thinned to 25 µm in thickness by mechanical exfoliation (due to its unique two-dimensional layer structure) to achieve the desired flexibility and transparency. However, the process still involves some complications due to multiple carriers and stack transfer.

In a flexible PMUT platform developed at IMEC, the fabrication was entirely carried out on a single temporary carrier (follow Fig. 3b for the fabrication schematic, Fig. 4e for the device schematics, and Fig. 5c shows the fabricated device images)^73^. They coated an epoxy resin on top of polyimide deposited on a glass substrate and patterned it by photolithography, to create the sidewalls of the membrane. This was followed by a polyimide film lamination on the top surface of the support layer using adhesive bonding. This is a passive layer on top of which electrodes and PVDF layers are realized. Finally, the first polyimide foil is delaminated from the carrier glass substrate, to enable flexibility. Carrying out all the processing on a single rigid temporary carrier wafer with a final release step is comparatively easier. This approach has been followed also in various reported top-down flexible PMUT processes. In a top-down process, layers are stacked in a reverse manner. The most critical part of the device, the membrane, is processed first followed by sidewall stacking, and can be released without having a separate PMUT substrate layer. Peter et al. introduced such an inverted flexible PMUT configuration (see Fig. 3d for the fabrication schematic and Fig. 4h for the device schematic)^74^. The transducers were monolithically fabricated on top of a thick polyimide substrate, which also formed a part of the membrane as a structural layer, on a glass carrier. The polyvinylidene fluoride-trifluoroethylene (PVDF-TrFE) layer was used as a piezoelectric material along with electrode patterning. To prevent the layer diffusion of photoresist into piezoelectric, a thin inorganic layer was introduced on the (PVDF-TrFE). After that, a thick photoresist is spin-coated on top to prevent excessive damping of the membrane vibration. The vibrating membrane is defined by an opening in the thick photoresist layer. After processing, the transducers were released with laser assistance or mechanically from the glass carrier. The release process can also be carried out using a sacrificial layer. Liu et al. developed a transfer process that provides large-area PZT thin films released from a high-temperature growth substrate to a few microns thick flexible polyimide substrates^75^. Using the same, the group demonstrated PMUTs fabricated on polyimide substrates using released PZT films (see Fig. 3d for fabrication schematic, Fig. 4f for the device schematic, and Fig. 5d for the images of fabricated devices)^76^. A ZnO sacrificial layer was used on a silicon wafer, whereas aluminum oxide (Al_2_O_3_) served as a diffusion barrier between ZnO and PZT. Next, polyimide was patterned to form cavity sidewalls (also serves as substrate) before the sacrificial layer release. Authors claimed that the released PZT films have superior electrical properties compared to films on rigid substrates due to substrate declamping. However, as the membrane consists of only the PZT and electrodes, lacking a structural layer, hence is fragile. Moreover, the resonance frequency is very low.

Joshi et al. reported a similar approach to fabricating flexible PMUT with the PZT films on a polyimide substrate (see Fig. 3d for the fabrication schematic, Fig. 4g for the device schematic, and Fig. 5e-left for the images of fabricated devices)^45^. Here as well, the ‘substrate last’ processing allows for the high-temperature PZT deposition steps before low-temperature polymer processing (for the structure). However, they added an extra polyimide passive layer which serves two purposes: 1) keeps the neutral axis outside PZT which is necessary to have sufficient bending moment required for the desired PMUT functioning^71^. 2) increases the resonance frequency and along with reduced membrane diameter resonance frequency is further increased to MHz range. Moreover, they released the PMUTs by etching silicon with DRIE. In another work, they experimented with the sidewall material and demonstrated the PMUT on the SU8 substrate (Fig. 5e-right)^77^. SU8 can provide a good compromise between stiffness (resonance frequency) and flexibility. SU8 can be patterned into high-aspect-ratio transparent structures using photolithography^78,79^. Hence, SU8 can serve as an alternative to polyimide which has been extensively used for flexible ultrasound transducers owing to its mechanical, dielectric properties and easier processing^80^. However, the drawback is its low tensile strength. As expected, the PMUT on SU8 showed a higher quality factor and sensitivity than the one on polyimide but at a cost of reduced flexibility. Another aspect of this research was the 8×8 fully addressed array design. The 2D arrays are sought after for their functionality to provide a 3D scan. However, electrical interconnect design is more challenging for high-density 2D array devices in comparison with 1D arrays. For instance, full addressing (the top electrodes are accessed individually with separate interconnects as shown in Fig. 5e-right, and the bottom electrode is common, which is a usual way for obtaining a signal with high SNR) in the 8×8 array design just described needs 64 interconnects. However, as the array size increases, such a scheme increases the number of interconnects (e.g., 32*32 design would require 1024 interconnects for top electrodes) and could make interconnects unmanageable for arrays with an even larger number of elements.

To deal with a large number of elements, there are two commonly reported solutions: (i) multilayer interconnects, i.e. distributing top electrode interconnects over multiple interconnect layers separated by a dielectric which offers a promising strategy to tackle the interconnect problems^81^, however, compatibility of arrays having more than 1024 elements with ultrasound imaging systems is still a concern. (ii) the second solution is to use row-column addressing, which drastically reduces the number of interconnects (for an array with m numbers of rows and n columns, from m*n to m + n^82^. Recently, Joshi et al. reported a 30×12 element row-column addressed PMUT array based on PZT material (Fig. 5f)^83^.The same fabricated strategy as in Ref. ^45^ was employed with design modification. In this setup, the array is row-column addressed instead of fully addressed. Additionally, each element in this configuration comprises 4 PMUT cells connected in parallel. Along with the characterization as a transmitter as well as a receiver working at about 2 MHz, this study presented the device’s waterproof packaging by sealing the device with parylene. The findings exhibit notable advancements toward low-frequency imaging using flexible PMUT arrays. However, row-column configurations are susceptible to capacitive crosstalk, and their compatibility with ultrasound imaging systems is nontrivial. Nonetheless, the study demonstrated the device connection to electronics using anisotropic conductive films (ACFs). For flexible devices, rather than employing wire bonding or flip chip bonding, flexible flat cables (FFC) can be attached to the device using ACFs or adhesives as depicted in Fig. 5f. Low-temperature ACFs consist of adhesive polymer resin and conductive particles which increase the electrical conductivity^84,85^. Bonding and curing are done by application of optimal pressure and temperature. The electrical connections to the printed circuit board (PCB) are achieved typically by plugging in the FFC into a zero-insertion force (ZIF) connector on the PCB. Alternatively, the device can be directly bonded to ZIF connectors with ACF or soldering paste and FFC can be plugged into the other end of the ZIF connector^1^. However, the metal traces on FFC should have the same pitch dimension as the contact leads on the flexible sample for ACF bonding to work. Further, electronics in such ultrasound transducers is another vast topic and the reader is referred to other recent reports which demonstrate the basic electronics setup^86^ and its flexible implementation^19^.

#### Hybrid and meta-rigid approaches with silicon on insulator (SOI) fabrication process

SOI technology is becoming a mature and convenient PMUT fabrication technique, because of the easy realization of membranes with the already built-in passive silicon device layer. Hence the fabrication involves the deposition of active layers on the silicon device layer of the SOI wafer. The device layer also acts as the passive layer of the membrane. DRIE is performed on the patterned backside which etches the silicon handle completely to form PMUT membranes. Buried oxide acts as an etch stop. This is briefly demonstrated in Fig. 3e. Lee et al. demonstrated a hybrid approach for a flexible PMUT array^57^ using SOI PMUTs (device schematic in Fig. 4i and images of the fabricated device in Fig. 5g). Then the ultrasound transducer array was strongly bonded onto a PDMS substrate using an O_2_ plasma treatment and was precisely diced with a fixed pitch to achieve flexibility. However, mechanical dicing limited the element density. Moreover, it becomes difficult to interconnect array elements due to topographical discontinuity. Another shortcoming is that a 50 µm thick PZT sheet resulted in 80 Volts of the actuation voltage^57^. In contrast to this, Sadeghpour et al. demonstrated SOI flexible PMUTs with a PZT thin film and silicon springs structures (device schematic in Fig. 4j and images of the fabricated device in Fig. 5h)^64^. The silicon springs (acting as hinges) are fabricated by DRIE, and mechanical dicing was avoided. The arrays are electrically connected through metal tracks on top of the hinges. The proposed flexible PMUT array benefits from a high-performance PZT thin film with a small actuation voltage of the PMUT elements in comparison with the previous hybrid design and also demonstrates excellent flexibility. However, due to the introduction of springs and the DRIE process, the fill factor is compromised. The other challenge is that very small space (on the springs) is available for interconnects. This makes it difficult to access PMUTs individually and scale them up for applications such as biomedical imaging.

### Capacitive micromachined ultrasound transducer (CMUT)

**Fig. 6:**
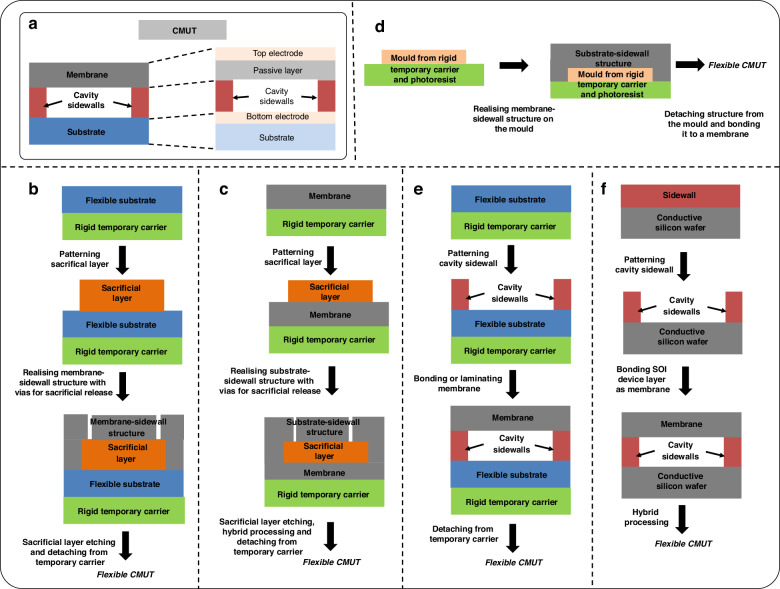
Overview of flexible CMUT fabrication. **a** Schematic of a CMUT^39,40^. **b** Sacrificial layer etching for cavity fabrication in CMUTs^3^. **c** Top-down fabrication for CMUTs with sacrificial layer for cavities^91^. **d** Top-down fabrication for CMUTs with molding the substrate with cavity sidewall^62^. **e** Bonding or lamination process^93^. **f** CMUTs with wafer bonding followed by a hybrid approach^2^. The schematics are not drawn up to scale

**Fig. 7:**
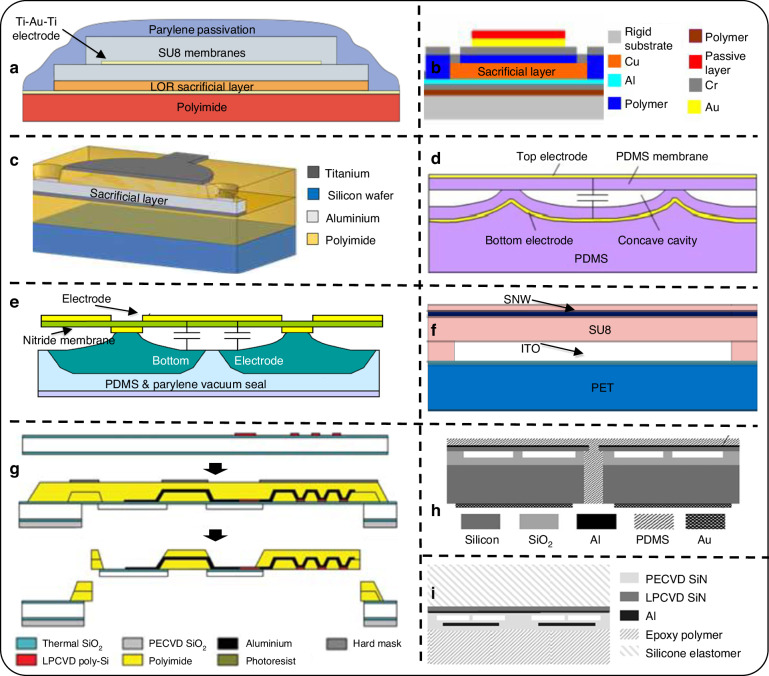
Overview of flexible CMUT designs. **a**–**c** Schematic of CMUTs realized using a sacrificial layer etching process^3,87,88^. **d**, **e** CMUTs with concave bottom electrodes^62,91^. **f** CMUTs using roll-lamination process^93^. **g**–**i** Hybrid approaches^2,51,95^. All pictures have been adapted with permission

**Fig. 8:**
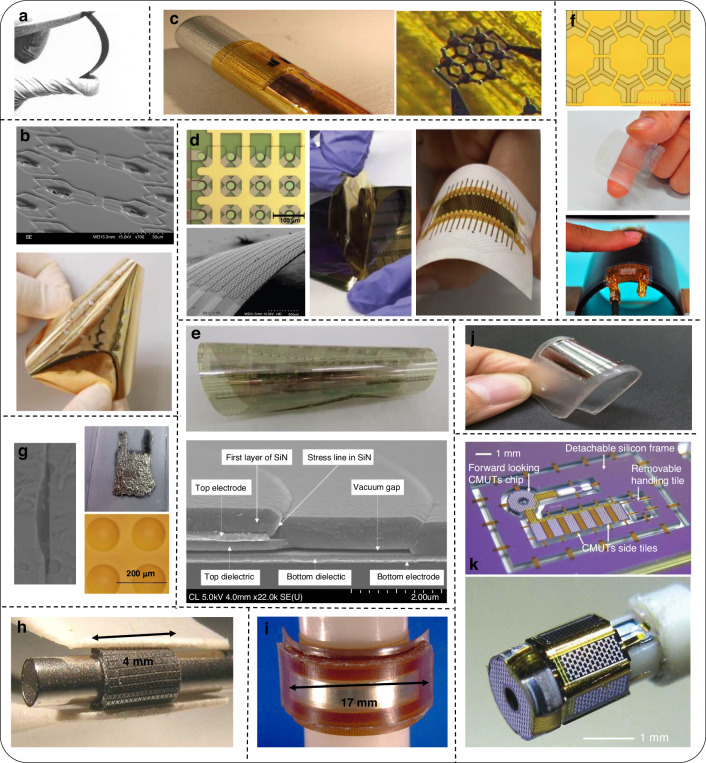
Overview of reported flexible CMUT devices. Sacrificial layer etched CMUTs. **a** CMUT with thinned-down silicon^49^. **b** SEM image of a polymer membrane with opened vias (top) and released ‘’Sonic Paper” CMUT arrays (bottom)^87^. **c** Polyimide-based CMUT wrapped on a cylindrical tube with a bending radius of 2.5 mm (left) and optical image of the curved CMUT with the 7 membranes of the test device, the membrane diameter being 50μm^88^. **d** Microscope (top left) and SEM (bottom left) images of ‘’polyCMUT” on a polyimide substrate, flexible substrate release from the silicon wafer carrier (middle), and optical images of released device on a flexible PCB (right)^3^. **e** Laser-released CMUTs on polyimide with SiN structural layer along with SEM image^90^. **f** SU8 CMUT realized using lamination process- Optical microscope image on the left, transparency shown in the middle, and flexibility on the right^93^. **g** CMUT on PDMS with a liquid metal electrode and concave cavity^62^. **h** Flexible silicon substrate realized by refilling through-wafer trenches with PDMS^2^. **i** Flexible CMUT array prototype realized by the hybrid reverse fabrication process being bent around a cylindrical bar with a radius of 8.5 mm^51^. **j** Row-column CMUT fabricated out of 2 bonded SOI waters^94^. **k** CMUT device attached in silicon frame using polyimide tabs (top). Forward and side-looking hybrid CMUTs at the tip of 2-mm diameter catheter^95^. All pictures have been adapted with permission

A CMUT element (Fig. 6a) has a capacitor structure. It consists of a thin metalized suspended membrane, over a cavity with a rigid metalized substrate. Typically, the membrane contains an insulation layer to prevent two electrodes from mutual contact. Upon applying a DC voltage across these electrodes, the membrane bends towards the substrate by electrostatic forces. Yet, the membrane’s inherent rigidity instigates a mechanical counterforce against this attraction. Consequently, ultrasound can be generated from the oscillations of the membrane with an AC voltage input. However, Qiu et al. point out that, in practice, high performance can only be achieved at a large DC bias near the collapse voltage, which increases the risk of failure of the device^71^. CMUTs can be operated in transmit as well as receive modes around their resonance frequency which depends on their membrane stiffness and gap^31^. The advantage of electrostatic CMUT technology is that the thin membrane can theoretically couple all the energy input to the acoustic domain because of no material-specific limitations and leads to high bandwidth as opposed to piezoelectric technology, where the coupling is limited by piezoelectric material properties like piezoelectric coefficient, stiffness, and dielectric constant^43,59^. It is worth noting that the CMUT membrane has a passive layer and top electrode, but the bottom electrode is attached to the substrate, which makes the separate substrate layer mandatory, unlike PMUTs. CMUTs are typically fabricated using either sacrificial layer release or bonding techniques. This is also reflected in flexible CMUT technology development as described below. The fabrication process flows are schematically shown in Fig. 6, while Figs. 7 and 8 present device schematics and images of reported flexible CMUTs, respectively.

#### Flexible substrate-based approaches

To the best of our knowledge, the first method used to manufacture a curved CMUT was based on wafer thinning to a range of approximately 50-150 µm to give flexibility to each of the CMUT dies (fabrication schematic in Fig. 6b and images of the fabricated device in Fig. 8a)^49^. After thinning and dicing the wafer to separate the transducer arrays, each flexible CMUT die can be placed inside a fixture through which the curvature is adjusted. The cavities were realized using sacrificial layer etching. The main drawback of this method is the limited radius of curvature that can be achieved. Moreover, the thin and flexible silicon-based dies can easily break during the assembly procedure, imposing severe handling precautions. This can be avoided with polymer-based CMUTs. In 2007, Chang et al. fabricated ‘sonic paper’ CMUT on a polymer substrate using sacrificial-layer techniques (fabrication scheme in Fig. 6b, device schematics in Fig. 7b, and images of the released device in Fig. 8b)^87^. By pre-patterning, hexagon islands that would be etched later to form final cavities were defined in the sacrificial (electroplated) Cu layer. Following this step, the cavity walls, called rails, and membrane structures were created by spinning polymer into the trenches between cavities and above the sacrificial layer, respectively. Finally, the sacrificial layer is etched to complete the fabrication. A very similar fabrication has been recently reported using only biocompatible materials- titanium for the electrodes and polyimide for the membrane and the backplate in the final device (schematics in Fig. 7c and images of the released device in Fig. 8c)^88^. The fabrication process was carried out on a silicon carrier with an aluminum sacrificial layer. However, the main drawback of typical polymer materials is the low breakdown field that makes it difficult to operate at voltages close to pull-in voltage. Lowering the pull-in voltage typically comes with lower resonance frequencies restricting use in high-frequency applications. This design optimization challenge was addressed by a new design for CMUTs encapsulating the electrode inside the membrane, instead of being on top which enabled low operational voltages and yet high frequencies comparable to traditional CMUTs^89^. Hence, the effective gap between the top and bottom electrodes (and therefore the operational voltage) was not dictated by the entire membrane thickness. This design allows the fabrication of low-frequency and high-frequency CMUTs with constant operational voltages (frequency does scale with the entire membrane thickness). A similar design with SU8 membranes (SU8 is being used as a structural material here) has been used for fabricating flexible ‘polyCMUTs’ with slight process modification (fabrication scheme in Fig. 6b, device schematic in Fig. 7a, and images of the released device in Fig. 8d)^3^. This process used lift-off resists (LOR) as a sacrificial layer for having the air gap and for the release of the flexible polyimide substrate from the carrier silicon wafer. This study towards musculoskeletal imaging presented advanced characterizations of the flexible CMUT including pulse-echo and durability while bending, and reliability for continuous operation. More recently, yet another process modification has been reported which uses inductively coupled plasma chemical vapor deposition (ICP-CVD) silicon nitride (SiN) structural layer instead of SU8 and laser lift-off for releasing the polyimide from the carrier (images of the fabricated device in Fig. 8e)^90^. During the fabrication, a critical point dryer is used after chromium (Cr) sacrificial release to avoid capillary stiction and the release holes are filled to form a vacuum-sealed cavity. This study presented scalable electrical characterization methods involving an impedance analyzer along with automatic probing and Python scripts to detect the pull-in voltage and resonance frequency, which can be used to assess yield and uniformity.

Another novel CMUT scheme used concave bottom electrodes increasing effective capacitance (fabrication schematic in Fig. 6c and device schematic in Fig. 7e)^91^. Theoretically, the effective capacitance can increase 10 times. In CMUTs, DC bias is applied to bring the membrane closer to the bottom electrode to increase its sensitivity. However, most of the developed CMUTs have flat-bottom electrodes, which cannot comply with the deflected membrane in a concave surface in the sense that only the 25% central area is more sensitive to the capacitance change (the other 75% of the area is considered as parasitic capacitance without coverage of the top electrode). For CMUT fabrication, they used a low- temperature process that only needs 80 °C with the SiN layer provided by the silicon wafer. The top-down processed scheme used a thermal reflow process which melts the patterned photoresist to form a spherical profile by surface tension. The concave nickel (Ni) bottom electrode is formed on top of the reflowed photoresist (sacrificial) in a concave spherical shape using the over-plating technique. Then, the photoresist was removed from the cavity through the release hole. The coating of PDMS and parylene later provided flexible substrate and vacuum-sealed cavities. The flexible CMUT structure is released from silicon using wet etching. Finally, the top electrodes are patterned to complete the fabrication. Note that stiffer materials like SiN (membrane) and electroplated nickel substrate are preferred for high-frequency applications. However, a downside of using SiN is the high residual stresses in the device leading to failure during handling. Shi et al. replaced SiN membranes with PDMS and followed the bonding approach (fabrication scheme in Fig. 6d, device schematics in Fig. 7d, and images of the released device in Fig. 8g)^62^. Also, liquid metal alloy electrodes were employed for stretchability. Layers of PDMS were bonded together by O_2_ plasma to complete the fabrication. The fabrication was carried out using two temporary glass substrates. Also note that, while dealing with flexible devices, the flexibility adds complexity to the electrodes and interconnect design as usual thin-film metal electrodes can experience cracks or delamination while bending. The use of liquid metal electrodes is one of the ways to delicately handle this aspect. However, there are a couple of other ways that could be borrowed from reported classical piezoelectric transducer technology. Kim et al. developed a flexible transducer with spray-coated stretchable electrodes^92^. The Ag nanowires were embedded below the surface of the PDMS elastomer to make the electrodes stretchable. This method also provides the required flexibility and low conductivity of conductive-polymer-type electrodes. Using wavy structures is another common strategy to avoid cracks in metal lines during a stretching operation. Jiang et al.^20^ pressed a Cu wire in a helical structure into a flat plane using a polished ingot to achieve flexible wavy electrodes. Another way is to use Laser ablation to pattern wavy electrodes and interconnects as in Ref. ^18^.

Pang et al. demonstrated a bonding scheme for transparent flexible CMUT using roll-laminated SU8 structures for the membrane (fabrication scheme in Fig. 6e, device schematics in Fig. 7f, and images of the released device in Fig. 8f)^93^. This polymer-based CMUT comprises an ITO-PET substrate where Indium tin oxide (ITO) is a bottom electrode, SU-8 sidewall, and vibrating membranes, and an Ag nanowire transparent (top) electrode. The CMUT was fabricated through a low-temperature roll-lamination technique. Lamination can be thought of as a wafer-bonding analogue for flexible CMUTs. In this process, SU8 was first spin-coated onto an ITO-PET substrate on a silicon temporary carrier and was patterned for sidewalls. Another SU8 layer was prepared on a PET release layer. The latter was roll-laminated as vibrating membranes onto the sidewall and was patterned. The PET release layer was then removed. For top electrodes, transparent silver nanowire electrodes were dip-coated and SU8 was employed for electrode protection. Finally, the wafer was removed to complete the fabrication. However, in this design, 140 µm diameter and 2 µm gap could result in a delicate membrane lamination process and affect the yield. Beyond this limitation, the CMUT has the advantage of transparency that provides unique functions in display panel applications.

#### Hybrid approaches

Zhuang et al. reported flexible CMUT arrays by forming polymer-filled deep trenches in a silicon substrate (fabrication scheme in Fig. 6f, device schematics in Fig. 7h, and images of the released device in Fig. 8h)^2^. This CMUT uses a vacuum cavity patterned in the oxide on a highly conductive silicon wafer. A SOI wafer is then fusion bonded to the prime silicon wafer. After removing the silicon handle and buried oxide layers from the backside of the SOI wafer, the remaining thin silicon device layer functions as a movable membrane and top electrode. The hybrid processing involves etching deep trenches using DRIE. The deep trenches are filled with PDMS, and the wafer is thinned down from the backside. The main limitation of this approach was the large area of silicon membrane regions overlapping the trenches, which resulted in some cracks after bending. Another work from Chen et al. can be thought of as an extension of the above idea (Fig. 8j shows the image of a curved array device). They developed a curved row-column CMUT array with a double conductive SOI wafer bonding process^94^. This process used wafer thinning using a wet etching process which removed handle layers from both wafers in separate steps. This allowed radius of curvature as small as 10 mm, locally. The thin silicon plates were encapsulated with PDMS and epoxy. Cavities are formed in the grown oxide from one of the SOI wafers whereas DRIE was used to etch out the trenches to isolate the electrodes. However, a large trenches width can cause thin layers to collapse and should be avoided when using the wafer thinning approach. Another hybrid curvilinear CMUT array design positioned the CMUT die as close as possible to the neutral surface to minimize strain on rigid parts of the die (fabrication scheme in Fig. 6c, device schematics in Fig. 7i, and images of the released device in Fig. 8i)^51^. This top-down fabrication process conveniently allows the etching holes (needed to realize the cavities) and the electrical pads to be on the non-radiating side. The process uses potassium hydroxide (KOH) etching to release the CMUTs from low-pressure chemical vapor deposition (LPCVD) SiN-covered silicon carrier. LPCVD SiN serves as a membrane material and plasma-enhanced chemical vapor deposition (PECVD) SiN for the structure. The cavity is made by etching the Cr sacrificial layer. After completion of the process, the wafer is diced and wire-bonded to a flexible circuit. A backing material, Eco gel, is then applied to the backside of the die using appropriate means, and the silicon is removed by DRIE. In the final step, a silicone elastomer, which has favorable properties as an acoustic lens material, is poured onto the membranes to serve as a coating and second encapsulating layer. The flex-to-rigid (F2R) platform reported in^95^ allowed for the fabrication of polymer links on rigid devices (schematics of the fabrication steps in Fig. 7g and images of the fabricated device in Fig. 8k). It promised the assembly of flexible sensors for minimally invasive medical instruments such as catheters and guidewires with tiny bending radii. Being a post-processing technology based on a ’polymer last’ approach, it can be applied to any preprocessed wafers. Hence devices fabricated on silicon wafers can be transferred onto polyimide and partially rendered flexible using a two-step backside silicon DRIE. It is even possible to scale the devices down to the dimension of the smallest guidewires. Although flexible polyimide links could improve the bending radius, this also involves a thinned-down substrate and associated challenges.

## Discussion, challenges, and outlook

Tables 1 and 2 provide a detailed overview of PMUTs and CMUTs, revealing the need for further development to meet specific application requirements and hence demonstrate proof-of-concept for applications. Table 3 qualitatively compares classical transducers and emerging MUTs. Although MUTs can be attributed to more design control, acoustic matching, small feature sizes and hence power consumption, batch fabrication, and CMOS integration, classical transducer technology still is a preferred choice for applications such as imaging or sensing due to higher acoustic pressures. To make the MUTs more competitive, efforts should be put into enhancing the flexible MUT acoustic pressures.

**Table 1:** Summary of flexible PMUTs

Substrate	Piezo material	Membrane Diameter (µm)	Membrane Thickness (µm)	Array Distribution	Pitch (mm)	Characterization studies	Frequency (kHz) (%bandwidth)	Sensitivity (nm/V)	Bending Radius (mm)	Proposed Application	Ref.
PI	PVDF	750	60	Single element with 3 ×3 cells		In-air transmit, receive function	161	~5	~5	Rangefinding	Liu^63^
PI	PVDF	700	80	Two element		Hydrophone, underwater pulse-echo	150 in water	9.3 Pa/V at 12.5 cm		ToF based sensing	Su^72^
PI	AlN	250	1.2			LDV, impedance	2580	30 (k = 1.42%)			Sun^66^
Polymer epoxy based	PVDF	480	16	64 ×64row-column	0.625	LDV, microphone	400	0.14 Pa/V at 1 cm		Haptics	Halbach^58^
PI	PVDF		20	300+ cells annular		LDV, dielectric, hysterisis	66	~2.4			Peters^74^
PI	PZT	400	1			Impedance, hysteresis	36.8				Liu^76^,
PI	PZT	100	7	30 ×12Row-column	0.5	LDV, hydrophone	2100 in water (20%)	40 Pa/V at 1 cm	8	Medical Imaging	Joshi, 2024
Silicon-PDMS	PZT	700	50	16 ×1	1.1	Impedance, hysteresis and hydrophone	700		5	Brain Stimulation	Lee^47^
Silicon	PZT	410	7	Single element with 3 ×3 cells	0.49	LDV	426 (1%)	4500	1		Sadeghpour^64^

**Table 2:** Summary of flexible CMUTs

Substrate	Gap (µm)	Membrane Diameter (µm)	Membrane Thickness (µm)	Array Distribution	Pitch (mm)	Characterization studies	Frequency (kHz) (%bandwidth)	Vop (V)	Bending Radius (mm)	Proposed Application	Ref.
Silicon				128 ×1	0.375	Underwater pulse-echo and phantom	4000 (125%) in water		40	**Demonstrated Abdominal imaging**	Wong^49^
Polymer-based	5	140	5	5 ×5 elements with 478 cells each	0.7	In-air pulse-echo	1000	120 V DC+16Vpp AC	2	Imaging	Chang^87^
PET	2	140	5	72 elements with 416 cells each	0.15	In-air pulse-echo	880	100 V DC + 300 Vpp AC	40	in display panels	Pang^93^
Ecogel Epoxy				192 ×1		Impedance, hydrophone, underwater pulse-echo	10700 (105%) in water		8.5	Endocavity imaging	Caronti^51^
PDMS	1	160	10	Single element with 2500 cells	0.2	Impedance	200				Shi^62^
PI	0.7	80	9.3	32 ×1 elements with 540 cells each		Impedance, hydrophone, underwater pulse-echo	4000 (75%)	50 V DC + 30Vpp AC	30	Musculoskeletal imaging	Omidvar, 2022
PI	0.35	50	2.5	Single element with 7 cells		LDV	4800	80 V DC, 30 nm/V	1	In implanted devices	Lucarini^88^
Silicon-PDMS	0.15	35*140 rectangular	1.83	16 ×16	0.25	Impedance	4500	70-100 V DC	0.45	Intravascular Ultrasound	Zhuang^2^
Silicon-PDMS	0.25	40	1.5	32 ×32 row-column	0.4	LDV, hydrophone	4500	60 V DC	10	Imaging	Chen^94^

**Table 3:** Classical vs micromachined ultrasound transducer

Factor under consideration	Classical Transducer	PMUT	CMUT
Design	Direct dependence of frequency on thickness limits its suitability for applications	More degrees of freedom associated with membrane and structural design lead to efficient designs	
Feature size	Limited by mechanical dicing	Small feature sizes defined by photolithography	
Acoustic matching	Complicated matching layers	No need of matching layers because of thin membrane	
Electronics integration	Involves conventional approaches	CMOS integration possible	
Fabrication process	Custom processing and poor yield	Batch processing	
Bendability	Limited by size of rigid piezoceramic islands	Intrinsically high for the designs on a flexible substrate	
Bandwidth	Low as a result of thick piezoceramic	Negatively affected by piezoelectric thin film properties	With appropriate DC bias, thin membrane results in high bandwidth
Sensitivity and SNR	High due to higher vibrating aperture and higher acoustic pressure	Low owing to reduced effective vibrating area and high	Bias dependent sensitivity, SNR affected by parasitics
Operation voltage	High due to bulk piezoelectric	Low due to energy-dense piezoelectric thin films	High owing to weaker electrostatic forces

In addition to the common advantages of micromachining technology, there are important distinctions between PMUT and CMUT in terms of bandwidth, sensitivity, SNR, and operating voltages. CMUTs generally exhibit higher bandwidths and electromechanical coupling compared to PMUTs, which are limited by material properties that restrict their electromechanical coupling. In the context of PMUTs, material innovation plays a crucial role in improving performance, with a specific focus on exploring crystal texture characteristics, piezoelectric composites, and lead-free materials. Moreover, it is essential to advance fabrication methods on an industrial level to incorporate a variety of piezoelectric materials alongside the commonly preferred AlN for cost-effective manufacturing. Piezoelectric technology benefits from the large energy density in the piezoelectric thin film, which results in high sensitivities with low operation voltage, and higher SNR, when compared with CMUTs.

In flexible MUTs, there is always a challenge in balancing flexibility and performance. While flexible approaches are more common, providing high bendability intrinsically, they require significant performance improvements. Moreover, while dealing with flexible substrates, residual stress in thin films can significantly affect the desired characteristics of MUTs, for instance, it can affect stiffness and in turn, alter resonance frequency and flexibility. This calls for in-depth research on residual stresses in thin films on flexible substrates. Hybrid approaches, such as devices with silicon-PDMS substrates, tend to offer better performance than completely flexible approaches. However, flexibility is partially compromised due to rigid components in hybrid designs. Meta-rigid approaches are less explored but hold potential for high-performance devices. Furthermore, both meta-rigid and hybrid approaches have not yet been sufficiently developed to incorporate the necessary interconnects and electronics integration aspects for ultrasound array operation. Research and development in this area could lead to the realization of intriguing applications. Whether utilizing electrostatic or piezoelectric technology, 2D arrays offer more intricate data for analysis. However, designing interconnects poses a challenge in these arrays. In this context, technologies such as row-column addressing, multilayer structures, or 3D integration could pave the way forward. Another obstacle with MUTs is their lower signal-to-noise ratio (SNR) compared to traditional transducers. In addition to enhancing acoustic pressures, addressing and minimizing crosstalk resulting from electrical and mechanical phenomena in different device designs and application environments can help tackle this issue. Additionally, examining the electrical impedance of the transducer and optimizing it to align with the electrical system impedance can also enhance the SNR.

Design goals for flexible ultrasound transducers include broad bandwidth, high sensitivity, cost-effective fabrication, seamless integration with electronics, and user comfort in biomedical applications. Through research on design-fabrication optimization, flexible PMUTs and CMUTs hold the potential for achieving these goals. Despite challenges, the field is driven by innovative technologies and dedicated researchers, offering opportunities for future applications and commercialization.
